# In Vitro and In Vivo Antifibrotic Effects of Fraxetin on Renal Interstitial Fibrosis via the ERK Signaling Pathway

**DOI:** 10.3390/toxins13070474

**Published:** 2021-07-09

**Authors:** Yi-Hsien Hsieh, Tung-Wei Hung, Yong-Syuan Chen, Yi-Ning Huang, Hui-Ling Chiou, Chu-Che Lee, Jen-Pi Tsai

**Affiliations:** 1 Institute of Medicine, Chung Shan Medical University, Taichung 40201, Taiwan; hyhsien@csmu.edu.tw (Y.-H.H.); kevin810647@gmail.com (Y.-S.C.); n735926688@gmail.com (Y.-N.H.); 2 Department of Medical Research, Chung Shan Medical University Hospital, Taichung 40201, Taiwan; 3 Division of Nephrology, Department of Medicine, Chung Shan Medical University Hospital, Taichung 40201, Taiwan; a6152000@ms34.hinet.net; 4 School of Medicine, Chung Shan Medical University, Taichung 40201, Taiwan; 5 School of Medical Laboratory and Biotechnology, Chung Shan Medical University, Taichung 40201, Taiwan; hlchiou@csmu.edu.tw; 6 Department of Medicine Research, Buddhist Dalin Tzu Chi Hospital, Chiayi 62247, Taiwan; turtle12062001@gmail.com; 7 School of Medicine, Tzu Chi University, Hualien 97010, Taiwan; 8 Division of Nephrology, Department of Internal Medicine, Dalin Tzu Chi Hospital, Buddhist Tzu Chi Medical Foundation, Chiayi 62247, Taiwan

**Keywords:** chronic kidney disease, fraxetin, indoxyl sulfate, α-SMA, ERK

## Abstract

Fraxetin, a natural derivative of coumarin, is known to have anti-inflammatory, anti-oxidant, and hepatoprotective effects in multiple diseases and in liver fibrosis. Whether fraxetin exerts similar effects against renal fibrosis is unknown. In a Unilateral Ureteral Obstruction (UUO) mouse model of renal fibrosis, fraxetin decreased UUO-induced renal dysfunction with a marked reduction in renal interstitial collagen fibers as detected by Masson’s Trichrome staining. Fraxetin treatment also inhibited the expression of α-SMA, Collagen I, Collagen IV, fibronectin, N-cadherin, vimentin, phosphorylated-ERK, and increased the expression of E-cadherin in UUO mice, as shown by immunohistochemical staining and western blot analysis. In vitro studies showed that fraxetin and indoxyl sulfate had no cytotoxic effects on MES13 kidney cells, but that fraxetin significantly decreased IS-induced cell motility and decreased protein expression of α-SMA, N-cadherin, vimentin, and Collagen IV via the ERK-mediated signaling pathway. These findings provide insight into the mechanism underlying fraxetin-induced inhibition of fibrogenesis in renal tissue and suggest that fraxetin treatment may be beneficial for slowing CKD progression.

## 1. Introduction

Chronic kidney disease (CKD) is one of the most common chronic health conditions in developed countries. The multiplicity of risk factors for CKD contributes to its high prevalence. CKD is a major health issue, with high co-morbidities and mortality. Further-more, many CKD patients are unaware of their condition until the disease has progressed to an advanced stage [1,2]. Regardless of the etiology of CKD, renal fibrosis represents the final pathway leading to end-stage renal disease [3]. As CKD progresses, uremic toxins accumulate and contribute to overall organ dysfunction [4]. Treatment strategies include medications to treat the primary disease, avoidance of nephrotoxic agents, and multidisciplinary team care [5,6]. The increasing prevalence of CKD necessitates the development of novel and effective therapies to slow its progression.

Evidence shows that chronic sustained renal damage leads to trans-differentiation of tubular epithelial cells, known as the epithelial-mesenchymal transition (EMT), eventually resulting in apoptosis and the deposition of fibrous tissue composed of collagenous extracellular matrix [7]. During the EMT, kidney tubule cells lose their epithelial characteristics and adopt a mesenchymal phenotype [8,9]. Studies show that blocking the trans-differentiation of renal tubule cells prevents EMT-induced inflammation, oxidative stress, and apoptosis by mediating transforming growth factor β (TGF-β) pathways [10,11,12]. Oxidative stress, inflammation, and the EMT are also caused by the uremic toxin indoxyl sulfate, a tryptophan metabolite that accumulates during CKD progression. In vitro and in vivo studies show that indoxyl sulfate upregulates the intrarenal renin angiotensin-aldosterone and mitogen-activated protein kinase (MAPK) pathways, resulting in renal fibrosis [13,14,15].

Fraxetin (7,8-dihydroxy-6-methoxy coumarin), a natural coumarin derivative from the bark of fraxinus rhynchophylla, has a variety of pharmacological properties. Fraxetin has been shown to exert anti-oxidative, anti-inflammatory, and anti-fibrotic effects [16], provide neuroprotection [17,18], and improve glucose control in diabetic rats [19,20]. Recent studies show that fraxetin inhibits the fibrotic process of hepatic damage induced by carbon tetrachloride [16,21] and ethanol [22] by modulating apoptosis and oxidative stress. Whether fraxetin exerts similar effects against fibrosis in CKD is unknown. This study investigates the histological and molecular effects of fraxetin in vivo in a Unilateral Ureteral Obstruction (UUO) mouse model and in vitro in MES13 kidney cells.

## 2. Results

### 2.1. Fraxetin Inhibits Renal Interstitial Fibrosis in UUO Mice

**Figure 1:**
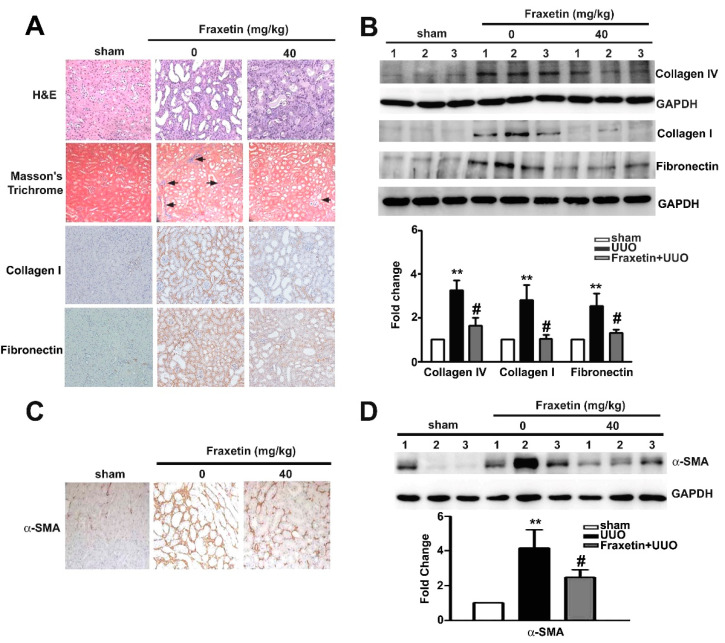
Effects of fraxetin on the morphological transformation and expression of collagen IV, collagen I, fibronectin and α-SMA in renal tissues of mice with unilateral ureteral obstruction (UUO). UUO Mice (*n* = 5) were oral gavage fraxetin (0 and 40 mg/kg) once daily for seven days and then sacrificed. (**A**) Morphological features of renal tissues visualized by hematoxylin and eosin (H&E) stain (upper panel), Masson’s Trichrome stain and immunohistochemistry assay. (**C**) α-SMA expression in renal tissues as determined by immunohistochemistry assay. (**B**,**D**) Western blot analysis of relative Collagen I, Collagen IV, fibronectin and α-SMA expression in total cell lysates of UUO tissues. GAPHD was used as an internal control for protein loading. Data are presented as the mean ± SE of at least three independent experiments. ** vs. sham surgery group; # vs. UUO group.

The Unilateral Ureteral Obstruction (UUO) mouse model is a well-established animal model of obstructive nephropathy and is characterized by the accumulation of collagen and increased extracellular matrix deposition during renal interstitial fibrosis progression [23]. Comparison of morphological features between UUO kidneys and fraxetin-treated UUO kidneys revealed that fraxetin significantly reduced renal tubule dilation (and) blue-stained interstitial collagen fibers, and decreased the expression of collagen I and fibronectin in the kidneys of UUO mice (Figure 1A). We also observed that fraxetin significantly decreased the collagen IV, collagen I, and fibronectin protein expressions in the kidneys of UUO mice compared to the UUO mice (Figure 1B). Consistently, fraxetin markedly reduced the accumulation and expression of α-SMA protein in the kidneys of UUO mice (Figure 1C,D). These results suggest that fraxetin improves renal function and reduces the damage and pro-fibrotic effects in UUO mouse kidneys.

### 2.2. Fraxetin Decreased the Expression of EMT-Related Proteins in UUO Mice

**Figure 2:**
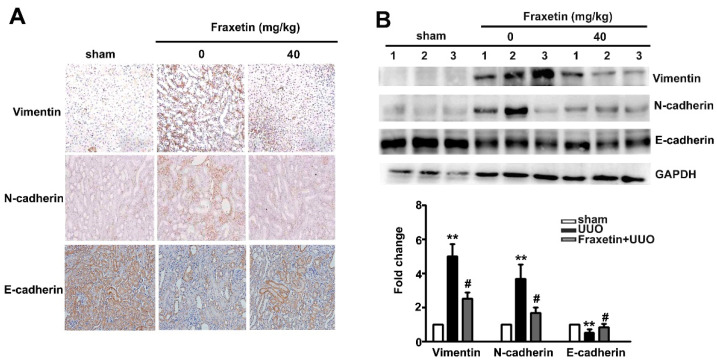
Effects of fraxetin on vimentin and N-cadherin expression in UUO mice. UUO mice (*n* = 5) were oral gavage fraxetin (0 and 40 mg/kg) once daily for seven days (**A**) Vimentin, N-cadherin and E-cadherin expression as determined by immunohistochemistry assay. (**B**) Western blot analysis of relative E-cadherin, vimentin, and N-cadherin expression in total cell lysates of UUO tissues. GAPHD was used as an internal control for protein loading. Data are presented as the mean ± SE of at least three independent experiments. ** vs. sham surgery group; # vs. UUO group.

The EMT plays a pivotal role in renal interstitial fibrosis progression [24]. To investigate the molecular mechanism whereby fraxetin inhibits renal fibrosis, IHC staining was used to examine E-cadherin, N-cadherin, and vimentin expression. Compared to the UUO mice, fraxetin (40 mg/kg) significantly decreased N-cadherin and vimentin expression and increased E-cadherin expression in the kidneys of UUO mice by IHC staining (Figure 2A). Western blot analysis showed decreased protein expression of N-cadherin and vimentin in the kidneys of fraxetin-treated UUO mice (Figure 2B). These observations indicate that the anti-fibrotic activity of fraxetin involves the regulation of EMT progression.

### 2.3. Effect of IS Combined with Fraxetin on Viability of MES13 Cells

**Figure 3:**
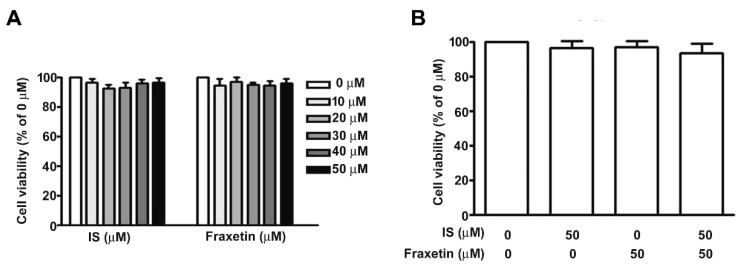
Effects of fraxetin and IS on MES13 cell viability. (**A**,**B**) MES13 cells were incubated with various concentrations (0, 10, 20, 30, 40, or 50 µM) of IS or fraxetin alone and or in combination for 24 h. MTT cell viability assay. Data are expressed as the mean ± SE of at least three independent experiments.

Next, we investigated the molecular mechanism underlying fraxetin-induced inhibition of renal fibrosis in vitro. Elevated plasma levels of IS are associated with chronic kidney disease progression in humans [25]. Treatment of MES13 cells with various concentrations of IS or fraxetin for 24 h had no dose-dependent effect on cell growth as determined by MTT assay (Figure 3A). Similarly, combination treatment with fraxetin and IS did not affect MES13 cell viability (Figure 3B).

### 2.4. Effect of Fraxetin on IS-Induced Motility of MES13 Cells

**Figure 4:**
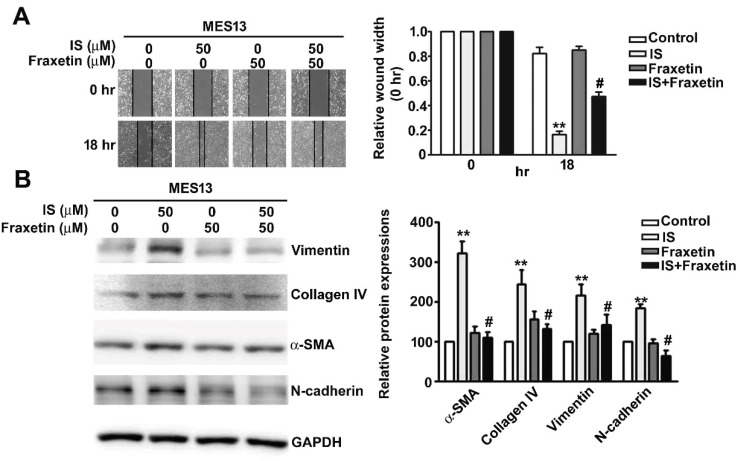
Effect of Fraxetin on IS-induced cell motility and fibrosis/EMT-related protein expression in MES13 cells. (**A**) MES13 cells were pre-treated with fraxetin (50 μM) for 2 h and then treated with IS (50 μM) for 18 h. Cell motility was determined by wound healing assay. At 0 and 18 h, cells were photographed under a light microscope at 400× magnification. (**B**) Total cell lysates were analyzed by western blot to determine the expression of vimentin, Collagen IV, α-SMA, and N-cadherin. GAPDH was used as an internal control for protein loading. Data are presented as the mean ± SE of at least three independent experiments. ** *p* < 0.01 vs. control (0 μM); # *p* < 0.01 vs. IS-treated cells.

Wound healing assays showed that MES13 cell motility was markedly increased by IS treatment. Pre-treatment of these cells with fraxetin significantly inhibited this increase in cell motility (Figure 4A). To investigate whether fraxetin inhibits IS-induced fibrosis in MES13 cells, Western blot analysis was used to determine the expression level of vimentin, Collagen IV, α-SMA, and N-cadherin after treatment with IS or fraxetin either alone or in combination. We observed that IS treatment increased the expression of α-SMA, Collagen IV, vimentin, and N-cadherin (Figure 4B). Cells treated with fraxetin before IS treatment exhibited lower expression of α-SMA, vimentin, and N-cadherin than did cells treated with IS alone (Figure 4B, Lane 4). Likewise, we used the human proximal tubule epithelial HK2 cell to suggest the similarly result of cell motility and western blotting (Supplementary Figure S1). In addition, we also demonstrated that fraxetin significantly inhibited TGF-β induced cell motility of human HK2 cells (Supplementary Figure S2A), as well as decreased the proteins expression of α-SMA, Collagen IV, vimentin, and N-cadherin by western blotting (Supplementary Figure S2B). These results indicated that fraxetin significantly inhibits fibrosis and EMT in IS-treated cells.

### 2.5. Effect of Fraxetin on Renal Fibrosis Involves ERK Phosphoryation Pathways in Vitro and In Vivo

**Figure 5:**
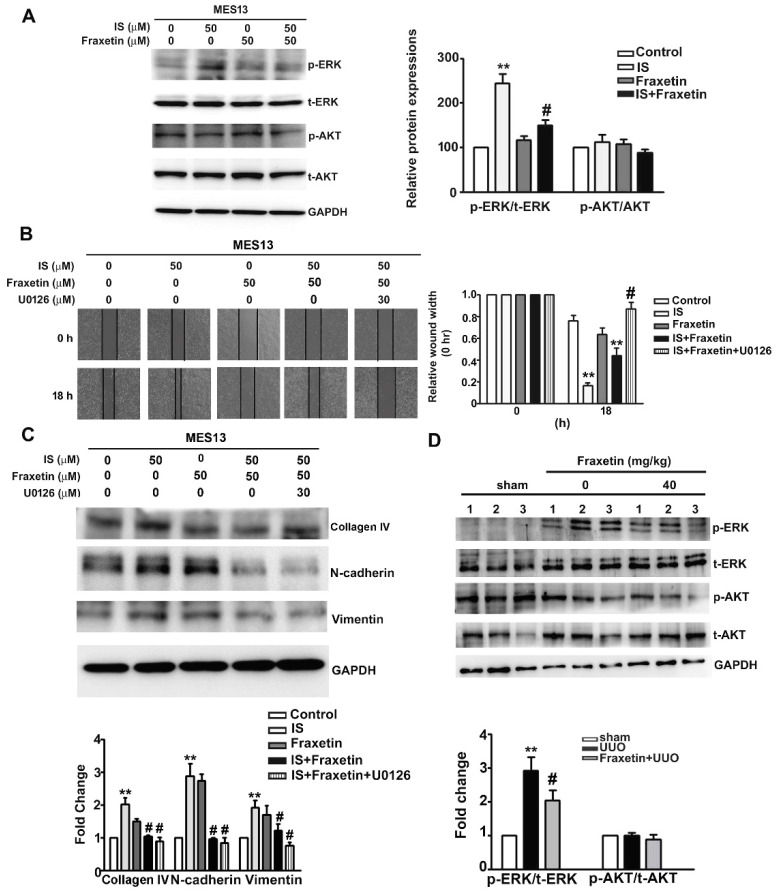
Fraxetin inhibited the ERK pathway activation in vitro and in vivo. (**A**) MES13 cells were pre-treated with fraxetin (50 µM) for 2 h and then treated with or without IS (50 µM) for 18 h. The expression levels of phosphorylated ERK (p-ERK), phosphorylated AKT (p-AKT), total-ERK (t-ERK), and total-AKT (t-AKT) were determined by western blot analysis. (**B**) MES13 cells were pretreated with MEK inhibitor (U0126; 30 µM) for 2 h and then cotreated with IS (50 µM) and/or fraxetin (50 µM) for 18 h. The cell motility was assessed by wound healing assay. (**C**) Collagen IV, N-cadherin, vimentin, and GAPDH expression was examined by western blotting. GAPDH was used as an internal control for protein loading. ** *p* < 0.01 vs. control; #, *p* < 0.01, compared with vs. IS alone or IS combined with U0126. (**D**) UUO mice (*n* = 5) were treated with fraxetin (0 or 40 mg/kg) for 7 days. The total protein in kidney tissue extracts was analyzed by western blot to determine the expression levels of p-ERK, t-ERK, p-AKT, and t-AKT. GAPDH was used as an internal control for protein loading. Data are presented as the mean ± SE of at least three independent experiments. ** *p* < 0.01 vs. sham surgery control or IS-treated cells; # *p* < 0.01 vs. UUO group.

The induction of renal fibrosis is known to involve activation of the ERK and AKT pathways [26]. To determine whether fraxetin affects the ERK and AKT pathways to inhibit IS-induced fibrosis, we compared protein expression between MES13 cells treated with IS alone (50 μM), fraxetin alone (50 μM), or IS with fraxetin pretreatment. Western blot analysis revealed elevated levels of phosphorylated-ERK in cells treated with IS alone. Pretreatment with fraxetin resulted in significantly lower levels of phosphorylated ERK but no significant difference in AKT phosphorylation (Figure 5A). In wound-healing assays, fraxetin/IS-treated MES13 cells incubated with the MEK inhibitor U0126 exhibited significantly lower motility than those without (Figure 5B). The motility of cells co-treated with fraxetin and U0126 was markedly reduced compared to those treated with fraxetin/IS (Figure 5B). In addition, U0126 significantly inhibited the protein expression of Collagen IV, N-cadherin, and vimentin in IS/fraxetin-treated cells (Figure 5C). The kidneys of UUO mice had significantly lower levels of phosphorylated-ERK than did control mice, with no significant difference in phosphorylated-AKT between UUO mice and controls (Figure 5D). These in vitro and in vivo findings indicate that fraxetin suppressed renal fibrosis progression by inhibiting activation of the ERK signaling pathway.

## 3. Discussion

From the literature, evidence had shown that chronic sustained injuries to the kidney result in apoptosis and morphological transformation of tubule epithelial cells characterized by upregulated expression of α-SMA and vimentin and dysregulated expression of E- and N-cadherin, which is known as the process of EMT [7]. Moreover, progressive worsening of renal function is associated with α-SMA–positive interstitial myofibroblasts in diabetic and membranous nephropathy [8,9]. Since EMT activation results in a vicious cycle of renal fibrosis that in turn induces more severe renal damage, several studies have shown that substances that block the process of trans-differentiation prevent EMT-induced fibrogenesis by mediating inflammation, oxidative stress, and apoptosis in renal tissues [12,27]. In gentamicin-induced nephrotoxicity, renal corpuscles and tubules pre-treated with resveratrol exhibit restored glutathione and catalase activity, decreased malondialdehyde content, increased E-cadherin expression, and decreased expression of α-SMA, TGF-β, and collagen [10]. The kidneys of UUO mice treated with ruxolitinib were observed to have lower levels of collagen deposition, α-SMA activation, TGF-β expression, inflammatory responses, malondialdehyde, and cleaved caspase-3 as well as elevated total superoxide dismutase arising from attenuation of ERK and Stat3 phosphorylation [11].Taken together, in this study we found that fraxetin had anti-fibrotic effects by decreasing the expression of α-SMA, collagen, N-cadherin and vimentin through down-regulation of the ERK signaling pathway. Although some evidences demonstrated that EMT may play an important role in renal fibrosis, EMT is still one of several mechanisms for renal fibrosis. It well known that myofibroblasts play a central role during kidney fibrosis [28]. When kidneys become injured, differentiation of pericytes are attached to peritubular capillaries and involved in basement membrane synthesis. Then, pericytes converse to myofibroblasts. This not only contributes to deposition of the extracellular matrix (ECM), but also regulates capillary rarefaction and inflammation, which leads to chronic kidney disease [29]. Recently, reports have suggested that endothelial cells can develop a myofibroblast phenotype in vitro and in vivo [30]. However, another report provided evidence that myofibroblast progenitors from interstitial pericytes in renal fibrosis, independent of endothelial cells, become myofibroblasts [31]. Further studies are needed to clarify the role fraxetin plays in this process (whether it is involved in interstitial pericytes or perivascular cells in myofibroblasts progression), to detect the specific myofibroblasts or pericytes marker, and to investigate these molecular mechanisms.

**Figure 6:**
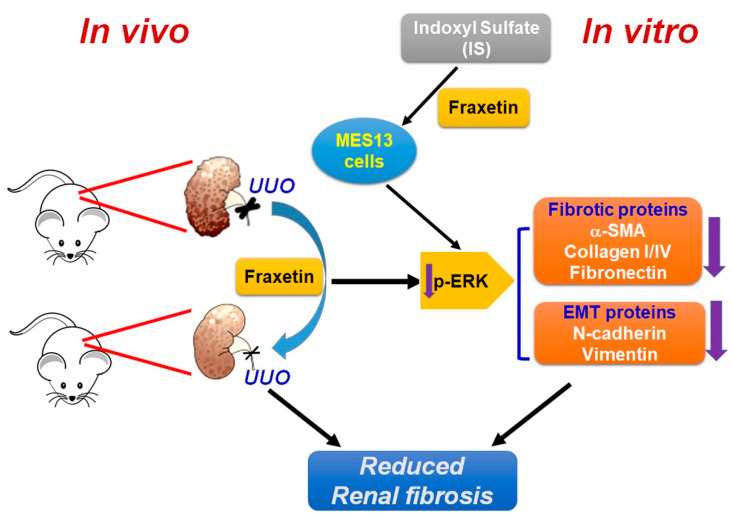
Schematic representation of the fraxetin-induced decrease in renal interstitial fibrosis via ERK signaling pathways both in vitro and in vivo.

As CKD progresses, IS accumulation induces fibrosis, inflammation, and oxidative stress, leading to renal dysfunction [13,14,15]. Evidence shows that IS induces cell transformation to a profibrotic phenotypic characterized by increased collagen and α-SMA deposition in renal tissues and upregulation of α-SMA, TGF-β, and collagen gene/protein expression in vitro through the heat shock protein 90 and Smad 2/3 pathways [13]. IS also induces inflammation-mediated tubuleinterstitial injury by upregulating the expression of monocyte chemotactic protein 1 to increase oxidative stress through activation of the ERK and JNK signaling pathways [32,33]. Being known as a uremic toxin, agents known to lower the serum levels of IS or to modulate the pathogenic process were hypothesized to have beneficial effects on renal functions [14,30,31]. An oral carbon absorbent was found to lower serum IS, ameliorate renal interstitial fibrosis, increase zonula occludens protein expression, and decrease α-SMA expression [34]. Medications such as angiotensin I receptor blocker and 1,25(OH)_2_D_3_ alleviate IS-induced phenotypic transformation by modulating the expression of TGF-β, Snail, and β-catenin through the Smads and PI3K/Akt signaling pathways, respectively [14,35]. Our study showed that fraxetin could inhibit the IS-induced fibrotic effects of MES13 and HK2 cells line as evidenced by suppressed motility ability, and inhibited expression of α-SMA, vimentin, and N-cadherin. Taken together, these findings support our hypothesis that substances that have anti-inflammatory or antioxidant effects, such as the fraxetin used in this study, act to decrease IS-induced EMT in renal cells (Figure 6).

Naturally-occurring free radical scavengers, such as fraxetin, could exert beneficial effects on the brain, liver, and kidneys by way of anti-oxidative, anti-apoptotic, or anti-fibrotic effects [16,22]. Studies of C57BL/6J mice treated with fraxetin showed marked antioxidative effects as evidenced by increased levels of superoxide dismutase, total and selenium-dependent glutathione peroxidase in brain tissues, increased glutathione reductase in hepatic tissues, and decreased oxidized/reduced glutathione in both brain and hepatic tissue [36]. Fraxetin pre-treatment of SH-SY5Y cells exposed to a mitochondrial inhibitor showed significantly decreased LDH release, intracellular peroxidase, lipid peroxidation, glutathione redox status, caspase-3 activity, and poly(ADP-ribose) polymerase cleavage, indicating that the neuroprotective effects of fraxetin involved decreasing apoptosis and free radical generation [18,37]. A series of studies of fraxetin on hepatic damage induced by carbon tetrachloride or ethanol revealed that fraxetin significantly increased the levels of anti-oxidant enzymes such as catalase, superoxide dismutase, and glutathione peroxidase and decreased inflammatory mediators such as tumor necrosis factor α and interleukin 1β to prevent hepatic fibrosis [16,22]. By using the UUO model in vivo and IS treated cells in vitro, the current study revealed that fraxetin ameliorated the progression of the EMT with the results of decreased collagen deposition as well as the EMT-associated proteins E-cadherin, α-SMA, vimentin, and N-cadherin.

Numerous studies have shown that substances which activated or inhibited the MAPK pathways, including ERK, SAPK/JNK, and p38, could mediate inflammation, apoptosis, proliferation, and differentiation, and ultimately regulate the process of fibrogenesis [38,39]. The anti-fibrotic agent ginsenoside Rb1 was shown to alleviate fibrotic effects in UUO mice and in HBSS-induced HK2 cells by inhibiting activation of autophagy through down-regulation of the AMPK/mTOR, ERK, and p38 signaling pathways [39]. Similarly, agents such as pirfenidone and renalase, were found to have anti-fibrogenic effects on renal tissue or HK2 cells, as evidenced by smaller areas of interstitial fibrosis, decreased α-SMA and collagen expression, and increased E-cadherin expression through modulation of the TGF-β1-mediated ERK, p38, and JNK signaling pathways [40]. Moreover, a study of carbon tetrachloride-induced hepatic damage revealed that fraxetin reduced the area-density percentage of collagen deposition in hepatic tissues and down-regulated the phosphorylation of p38 MAPK, JNK, and ERK, resulting in up-regulation of Bcl-2 and down-regulation of Bax, cleaved caspase-3, α-SMA, and vimentin, along with accelerated degradation of the extracellular matrix [16]. Contrarily, mice treated with arecoline, a well-known nephrotoxic substance, exhibited marked renal fibrosis together with the elevated expression of TGF-β, fibronectin, and plasminogen activator inhibitor 1 [41]. HK2 cells treated with arecoline displayed markedly increased mesenchymal-like properties, with decreased expression of E-cadherin, increased expression of N-cadherin, vimentin, α-SMA, and collagen through the ERK signaling pathway [27]. Taken all these studies together, we observed that fraxetin exerted anti-fibrotic effects by mediating EMT progression through down-regulation of the ERK signaling pathway.

In conclusion, our study showed that fraxetin exerts anti-fibrogenic effects, including decreased morphological transformation, lower cell motility, and decreased ECM synthesis on renal tissues through inhibiting the ERK signaling pathway.

## 4. Materials and Methods

### 4.1. Cell Lines, Reagent and Chemical Drug

Mouse glomerular mesangial cells (MES13) were provided by Dr. Chien-Chun Li (Department of Nutrition, Chung Shan Medical University) and HK2 cells were cultured in Dulbecco’s Modified Eagle Medium (DMEM)/Nutrient Mixture F-12 with 10% fetal bovine serum (FBS) and 1 × penicillin-streptomycin in a humidified atmosphere incubator with 5% CO_2_ at 37 °C. Fraxetin (purity ≥ 98%) was purchased from Chemface company (Wuhan, China). U0126 and indoxyl sulfate (IS) were purchased from Sigma-Aldrich (St. Louis, MO, USA).

### 4.2. Cell Viability Assay

Cell viability was determined by MTT assay. Briefly, MES13 cells were incubated in 24-well plates (5 × 10^4^ cells/well) for 24 h and then treated with various concentrations of IS (0~50 μM), fraxetin (0~50 μM), or both for 24 h. After incubation in MTT reagent (final concentration, 0.05 mg/mL) for 4 h, the medium was removed and the cells were treated with isopropanol and incubated for 15 min. The final absorbance was measured at 570 nm using an enzyme-linked immunosorbent assay (ELISA) reader. Cell viability was determined by the absorbance relative to that of the DMSO group.

### 4.3. Cell Motility Assay

MES13 cells (2 × 10^5^ cells/well) or HK2 cells (4 × 10^5^ cells/well) were seeded in 6-well plates and incubated to ap-proximately > 90% confluence. A wound was scratched into the cell layer using a 200-µL pipette tip, and the proliferation inhibitor mitomycin c (2 μg/mL) was added in serum free DMEM/F12 medium. After incubation for 18 h, suspended cells were washed off with serum-free medium. Cell motility was analyzed at 0 and 18 h using an inverted microscope. The average cell motility rate was calculated as the wound width / time.

### 4.4. Western Blotting

All procedure of immunoblotting assay was followed as previously reports [42]. The total proteins in cell lysates and kidney tissues were extracted, placed in a NETN buffer (20 mM Tris, 1mM EDTA, 150 mM NaCl, and 0.5% NP-40), and incubated in a cocktail (Pierce Biotechnology, Rockford, IL, USA). The protein concentration of each sample was determined using the Braford assay. Equal amounts of protein (20 μg) were separated via 8 or 10% SDS-PAGE for 2 h and transferred to polyvinylidene difluoride (PVDF) membranes for 1.5 h. The membranes were then incubated in blocking buffer (5% nonfat milk) for 1 h at room temperature. The membranes were incubated overnight at 4 ˚C with primary antibodies against α-SMA, collagen IV, collagen I, E-cadherin, fibronectin, N-cadherin, vimentin, p-ERK, t-ERK, p-AKT, t-AKT, and GAPDH. The membranes were washed for 5 min in a TBST buffer three times and incubated with a secondary antibody (HRP-labeled goat anti-mouse IgG or goat anti-rabbit IgG) for 1 h at room temperature. Band intensities were determined using the ECL detection reagent (Millipore, Darmstadt, Germany) and the LAS-4000 mini luminescent image analyzer.

### 4.5. Animal Model and Experimental Procedure

Male C57BL/6 mice (body weight, 22 g) were purchased from the National Labor-atory Animal Center (Taipei City, Taiwan). The Unilateral Ureter Obstruction (UUO) mouse model and experimental produces were used as described previously [43]. Renal fibrosis was induced in mice by UUO, and the mice were observed and measured for seven days. Twenty mice were randomly assigned into four groups: sham-surgery (*n* = 5), UUO (*n* = 5), UUO plus fraxetin (40 mg/kg) (*n* = 5), and UUO plus fraxetin (0 or 40 mg/kg). Fraxetin was administered via oral gavage once daily for seven days. After seven days, the mice were sacrificed and the kidneys were excised. The proteins were extracted from the kidney tissue in NETN buffer and analyzed by western blotting. In addition, kidney tissue samples were fixed in 10% formaldehyde and paraffin embedded for immuno-histochemical and histological analysis. Tissue samples were observed via microscopy and photographed. This study meets the ethics approval code 2420.

### 4.6. Immunohistochemistry Assay and Masson’s Trichrome Staining

The tissue samples were mounted on glass slides and incubated with an antibody against α-SMA (1:100), collagen I (1:200), fibronectin (1:200), E-cadherin (1:100), N-cadherin (1:100), or vimentin (1:200) in a dilution buffer and then with secondary antibodies for 1 h. Masson’s Trichrome staining was carried out as described for H&E staining to detect the interstitial collagen fibers of renal fibrosis.

### 4.7. Statistical Analysis

All data are presented as the mean ± SE. Analysis of variance (ANOVA) and an unpaired 2-tailed Student’s t-test was used to determine the significance of differences, and *p* < 0.01 or *p* < 0.05 was considered statistically significant.

## 5. Conclusions

These findings indicate that fraxetin exerts renoprotective effects that might be promising as an adjuvant therapy for CKD patients. Further research is needed to clarify details of the mechanism underlying the anti-fibrotic effects of fraxetin.

## Data Availability

The authors will freely release all data underlying the published paper upon direct request to the corresponding author.

## Supplementary Materials

The following are available online at https://www.mdpi.com/article/10.3390/toxins13070474/s1, Figure S1: Fraxetin inhibit IS-induced cell motility and fibrosis/EMT-related protein expression in HK2 cells, Figure S2: Fraxetin inhibit TGF-β-induced cell motility and fibrosis/EMT-related protein expression in HK2 cells.
