# Supplementary material for: In Vitro and In Vivo Antifibrotic Effects of Fraxetin on Renal Interstitial Fibrosis via the ERK Signaling Pathway
Source: Toxins (Basel). 2021 Jul 9;13(7):474. doi: 10.3390/toxins13070474 (PMC8310265; doi:10.3390/toxins13070474)
Supplement: Supplementary file 1 [file toxins-13-00474-s001.zip › toxins-1206226-supplementary.pdf]

Supplementary Materials: In Vitro and in Vivo Antifibrotic Effects of Fraxetin on Renal Interstitial Fibrosis via the ERK Signaling Pathway

Yi-Hsien Hsieh, Tung-Wei Hung, Yong-Syuan Chen, Yi-Ning Huang, Hui-Ling Chiou, Chun-Wen Cheng, Chu-Che Lee and Jen-Pi Tsai

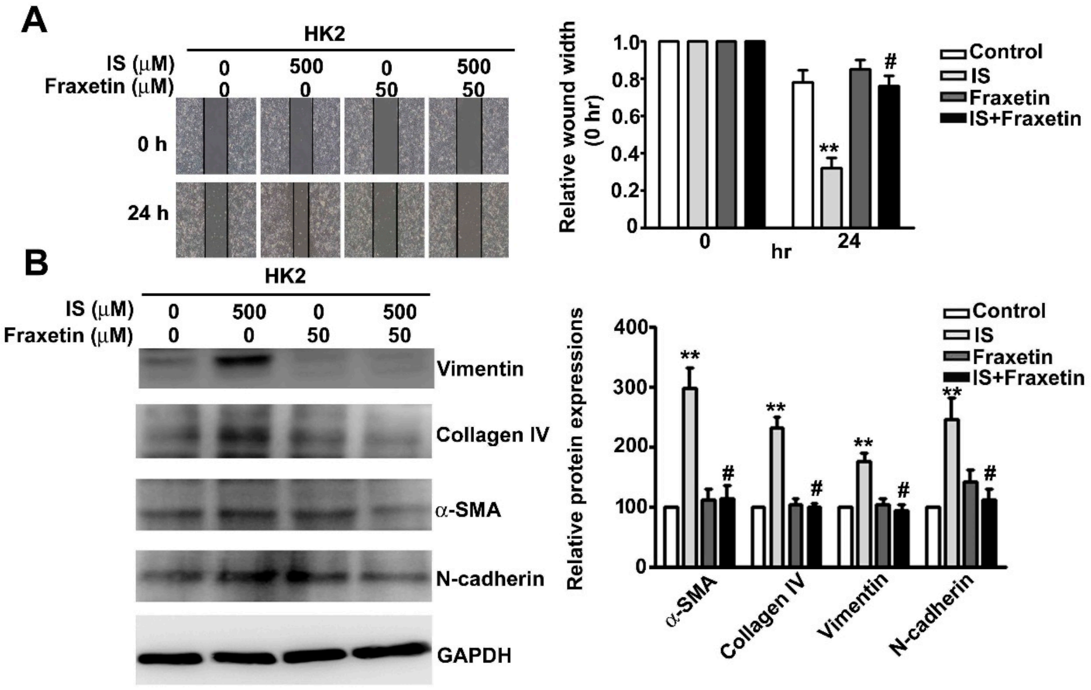

Figure S1. Fraxetin inhibit IS-induced cell motility and fibrosis/EMT-related protein expression in HK2 cells. (**A**) HK2 cells were pre-treated with fraxetin (50 M) for 2 h and then treated with IS (500 M) for 24 h. Cell motility was determined by wound healing assay. Cells were photographed under a light microscope at 400× magnification. (**B**) Total cell lysates were analyzed by western blot to determine the expression of vimentin, collagen IV, α-SMA, and N-cadherin. GAPDH was used as an internal control for protein loading. Data are presented as the mean ± SE of at least three independent experiments. ** $p < 0.01$ vs. control cells; # $p < 0.01$ vs. IS-treated cells.

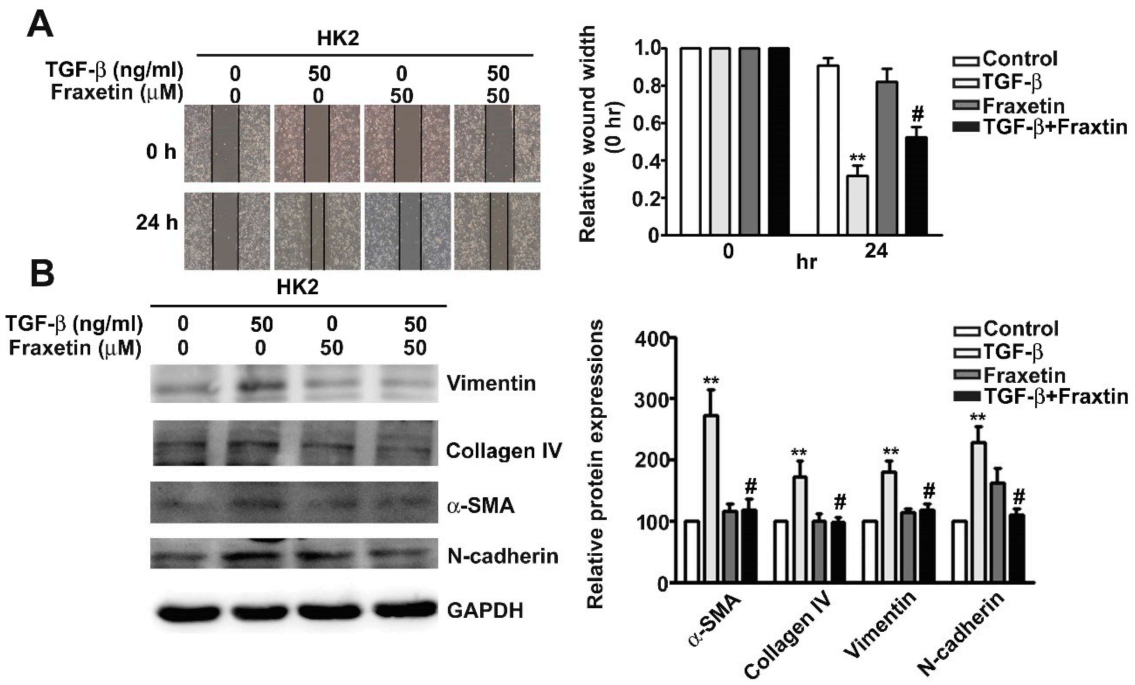

Figure S2. Fraxetin inhibit TGF-β-induced cell motility and fibrosis/EMT-related protein expression in HK2 cells. (**A**) HK2 cells were treated fraxetin (50 μM) for 2 h, then with or without TGF-β (50 ng/ml) treatment. Cell motility were showed by using the wound healing assay and photographed under a light microscope at 400× magnification. (**B**) The expression of vimentin, collagen IV, *α*-SMA, and N-cadherin were detected by western blotting. GAPDH was used as an internal control for protein loading. Data are presented as the mean ± SE of at least three independent experiments. ** $p < 0.01$ vs. control cells; # $p < 0.01$ vs. TGF-β-treated cells.
